# An Evaluation of the Genetic Structure of Geese Maintained in Poland on the Basis of Microsatellite Markers

**DOI:** 10.3390/ani9100737

**Published:** 2019-09-27

**Authors:** Joanna Warzecha, Maria Oczkowicz, Dominika Rubis, Agnieszka Fornal, Tomasz Szmatoła, Monika Bugno-Poniewierska

**Affiliations:** 1 Department of Molecular Biology of Animals National Research, Institute of Animal Production, 32-083 Kraków, Poland; maria.oczkowicz@izoo.krakow.pl (M.O.); dominika.rubis@izoo.krakow.pl (D.R.); agnieszka.fornal@izoo.krakow.pl (A.F.); 2 Centre of Veterinary Medicine, University of Agriculture in Kraków, 30-059 Kraków, Poland; tomasz.szmatola@izoo.krakow.pl; 3 Faculty of Animal Breeding and Biology Agricultural University in Cracow, 31-120 Kraków, Poland; monika.bugno@izoo.krakow.pl

**Keywords:** goose, conservative flocks, microsatellite markers, genetic diversity, structure analysis

## Abstract

**Simple Summary:**

This study was conducted with the aim of evaluating the genetic variability of Polish national goose breeds, using polymorphism of 15 microsatellite markers. The results revealed a highly mixed genotype of all the examined geese, suggesting that breeds cannot be distinguished from each other on the basis of microsatellite markers.

**Abstract:**

The aim of this study was to evaluate the genetic variability of the White Kołuda^®^ goose and 12 conservative flocks: Kielecka, Podkarpacka, Garbonosa, Pomerian, Rypinska, Landes, Lubelska, Suwalska, Kartuska, Romanska, Slowacka, and Kubanska, maintained in Poland using microsatellite data. The genetic diversity of geese kept in Poland remains poorly analyzed at the molecular level. In total 392 samples were examined with the usage of 15 microsatellite markers. 119 alleles were identified and the number of alleles per locus ranged from 1 to 13. The highest number of alleles was observed in TTUCG5 (16) and the lowest in CAUD-G007 (2), while CKW47 was monomorphic. The lowest value of expected heterozygosity (H_e_) was observed in Landes, while the highest in Romanska. Similarly, the observed heterozygosity (H_o_) was the lowest in Landes but the highest in Kartuska. The polymorphism information content (PIC) indicates loci TTUCG5 as the most valuable microsatellite marker among those examined. The Structure software was used for the first time to identify goose populations, revealing high admixture between breeds and their close genetic propinquity. Moreover, the presented panel of microsatellite markers remained polymorphic and is useful for population studies of geese and assessment of genetic diversity.

## 1. Introduction

Nowadays, monitoring of the genetic diversity of many species is based mainly on analyses obtained by advanced molecular methods. The information about population structures and variety is crucial to choose an appropriate conservation and management breeding program for species maintained in each country [1]. Anseriformes is a well-known and varied bird order revealing worldwide plurality as well as morphological and biological diversification [2]. The taxonomy of Anseriformes is complicated due to the close relationship between about 150 species included in the order; nevertheless, it is one of the best examined avian groups [3]. The most numerous and significant, from an economic perspective, continues to be the Anserinae family in which geese are classified, constituting one of the oldest domestic poultry, providing healthy meat, giblets, and feathers. Evolutionary goose species have been selected by natural and artificial selection [4]. Conservative breeds are a reservoir of valuable genes; thus they are valuable material for evolutionary research and biodiversity programs [5]. The preservation of conservative flocks’ genome is one of the priorities in goose breeding and may improve unique genetic traits. In Poland, there are 14 goose breeds included in the national program for protection of animal genetic resources [6]. Customarily, geese could be divided into their morphologic traits like body weight (heavy and light geese) or plumage color (unicolored or spotted). Moreover, the Polish White Kołuda^®^ goose is an economically important agricultural breed and excellent local goose variety. This breed represents 90% of the goose population bred in Poland. It was selected to enhance meatiness and reproduction traits, but also to improve resistance to diseases [4]. The meat of the White Kołuda^®^ goose is consumed worldwide, while the plumage is commonly used in production of high quality clothes, pillows, and duvets. At the same time, it constitutes a common object of falsification during production.

The genetic variability of domestic poultry based on DNA markers has been described by several papers, but generally avian species exhibit a low level of divergence at the molecular level [7]. Microsatellite markers have been evaluated in the greylag goose (*Anser anser*) [8], Canada goose (*Branta canadensis L.*) [9], swan goose (*Anser cygnoides L.*) [10], white-fronted goose (*Anser albifrons*) [11], and pink-footed goose (*Anser brachyrhnchus*) [12]. Moreover, microsatellite loci panel have been tested in a few Chinese breeds [10,13,14,15], the white Roman goose [15], Hawaiian goose [16], and Slovak domestic goose breeds [17], which also proved to be useful for estimating genetic diversity, resolving phylogenetic relationships between closely related populations, and genetic mapping [14,18,19]. The microsatellite loci analysis is widely used in population genetic studies of numerous poultry species and could be utilized as an estimator of population structure [20,21]. Among Polish local breeds, the genetic diversity of the Zatorska, Lubelska, Kielecka, Sub-Carpathian, Hunched Beak, Kartuska, Rypinska, Suwalska, and Pomeranian breeds has been well investigated based on microsatellite markers [5,7]. The aim of our study was to evaluate the genetic diversity of Polish breeds of geese by microsatellite DNA polymorphism.

## 2. Materials and methods

### 2.1. Animals and DNA Isolation

The experimental material was collected from the White Kołuda^®^ goose and 12 conservative geese flocks: Kielecka (Ki), Podkarpacka (Pd), Garbonosa (Ga), Pomerian (Po), Rypinska (Ry), Landes (La), Lubelska (Lu), wSuwalska (Su), Kartuska (Ka), Romanska (Ro), Slowacka (Sl), and Kubanska (Ku), maintained in the National Research Institute of Animal Production, Department of Water Fowl Breeding in Kołuda Wielka and Dworzyska. Ethical approval is not needed for research on plumage samples, while sourced blood was collected during routine slaughter of geese in Kołuda Wielka (statutory activity No. 04-011.1).

The DNA was isolated from 300 plumage samples (calamus) (22 individuals per breed of conservative flocks of geese and 36 individuals per White Kołuda^®^ goose) and additionally from 96 blood samples of the White Kołuda^®^ goose (in total 396 samples). DNA isolation was performed with the use of the Sherlock AX Isolation KIT (A&A Biotechnology, Gdynia, Poland) according to the instructions provided in the protocol.

### 2.2. Microsatellite DNA Amplification

The DNA polymorphism was assessed at 15 microsatellite loci (Bca µ1, TTUCG5, CKW21, Bca µ9, Bca µ8, Ans02, Ans18, Ans25, CAUD-G013, CAUD-G007, CKW47, Aal µ1, Afa35, CAUD-G012, Ans07) and analyzed by PCR reaction using labelled primers with one of four dyes: 6-FAM, VIC, NED, and PET, presented in Table 1.

The multiplex PCR was carried out on 20 ng of genomic DNA in two multiplex sets of primers and performed on a Veriti Thermal Cycler (Applied Biosystems, Foster, CA, USA) (Mix 1 (7plex): Bca µ8, Ans02, Ans18, Ans25, Aal µ1, CAUD-G012, Ans07; Mix 2 (8plex): Bca µ1, TTUCG5, CKW21, Bca µ9, CAUD-G013, CAUD-G007, CKW47, Afa35). The amplification protocol comprised initial denaturation and enzyme activation at 95 °C (5 min), followed by 29 cycles of denaturation at 95 °C (30 sec), primer annealing at temperature 55 °C and 58 °C (3 min), extension at 72 °C (30 sec), and final extension at 72 °C (5 min). Afterwards, products of PCR were separated in polyacrylamide gel performed on a 3130xl Genetic Analyzer (Applied Biosystems, Foster, CA, USA). The allelic sizes of all loci were estimated relative to the in-line 500 LIZ Size Standard marker. The results were genotyped in GeneMapper 4.0 (Applied Biosystems, Foster, CA, USA).

### 2.3. Statistical Analysis

GenAIEx 6 software was used to obtain allele frequencies, standard diversity indices (N, Na, Ne), as well as observed (H_o_) and expected heterozygosity (H_e_) for mentioned loci and populations of geese. Moreover, we used it to compute the fixation index (F_ST_). Departures from the Hardy–Weinberg equilibrium (HWE) were estimated in GENEPOP 4.2, while Arequin 3.11 software was used to calculate analysis of molecular variance (AMOVA) in order to evaluate the genetic variance in the populations and between them. Our own statistical program IMGBOVSTAT IZOO PIB was used to calculate polymorphic information content (PIC), estimated according to Botstein et al. [22]. The Structure software was used to identify the population structure and pattern of admixture within populations. Analysis was replicated 10 times per K, from K = 1–16 and performed with 200,000 iterations and 100,000 burn-in period. Structure Harvester [23] was used to compute ΔK statistics, while CLUMPAK and Distruct aligned the cluster membership coefficients of Structure runs and exhibited the results. The genetic distances between the breeds was estimated according to Nei et al. [18]. A principal component analysis (PCA) was performed among goose breeds with R.

## 3. Results

The analysis of 15 microsatellite loci showed CKW47 as monomorphic in investigated individuals—according to which, we analyzed 14 polymorphic; therefore, this locus was excluded from the analysis. Within the remaining 14 microsatellite sequences, 119 alleles were recognized and ranged from 3 to 13 in different alleles per locus in 392 examined geese. The highest number of alleles was observed in TTUCG5 loci (16) and CKW21 (15) and the lowest in CAUD-G007 (3). The population of White Kołuda^®^ geese and Romanska had the highest average number of alleles per locus (6.857 and 5.000 respectively), whereas Garbonosa (2.714) and Rypinska (2.714) were the lowest (Tables S1 and S2).

The genetic statistics relating to polymorphism were calculated to estimate the allelic diversity at each locus for every examined population. The average number of alleles per locus (Na) was 3.941 and the average number of effective alleles (Ne) per locus ranged from 1.800 (Rypinska) to 3.041 (Romanska). The mean value of observed heterozygosity was 0.361 per tested goose breed, the highest H_o_ was observed for Kartuska (0.479) and Romanska (0.415), while the lowest was computed for Landes (0.280). Romanska (0.638) and Kartuska (0.620) showed the highest values of H_e,_ whereas Landes (0.305) has the lowest value of this coefficient. The average value of H_e_ was 0.447 in the analyzed population (Table 2). The null alleles were not calculated due to no descent data.

The F_ST_ values for each locus are shown in Table 3 while the F_ST_ values for examined populations are shown in Table 2. The mean fixation index estimated over all populations for each locus was 0.122 and the value of fixation index varied from 0.061 (Aal) to 0.300 (Ans18). The highest F_ST_ was observed in Landes (0.080) and the lowest in Romanska breed (0.068). PIC values obtained in this study ranged from 0.165 (CAUD-G007) to 0.813 (TTUCG5) with an overall average of 0.463 (Table 4).

No deviations from HWE of examined microsatellite loci across the population were detected.

According to AMOVA analysis, 92.5% of the total genetic variance was distributed within populations (*p* < 0.05) and 7.5% was distributed among them. In AMOVA analysis we obtained a fixation index amounting to 0.075.

**Figure 1:**
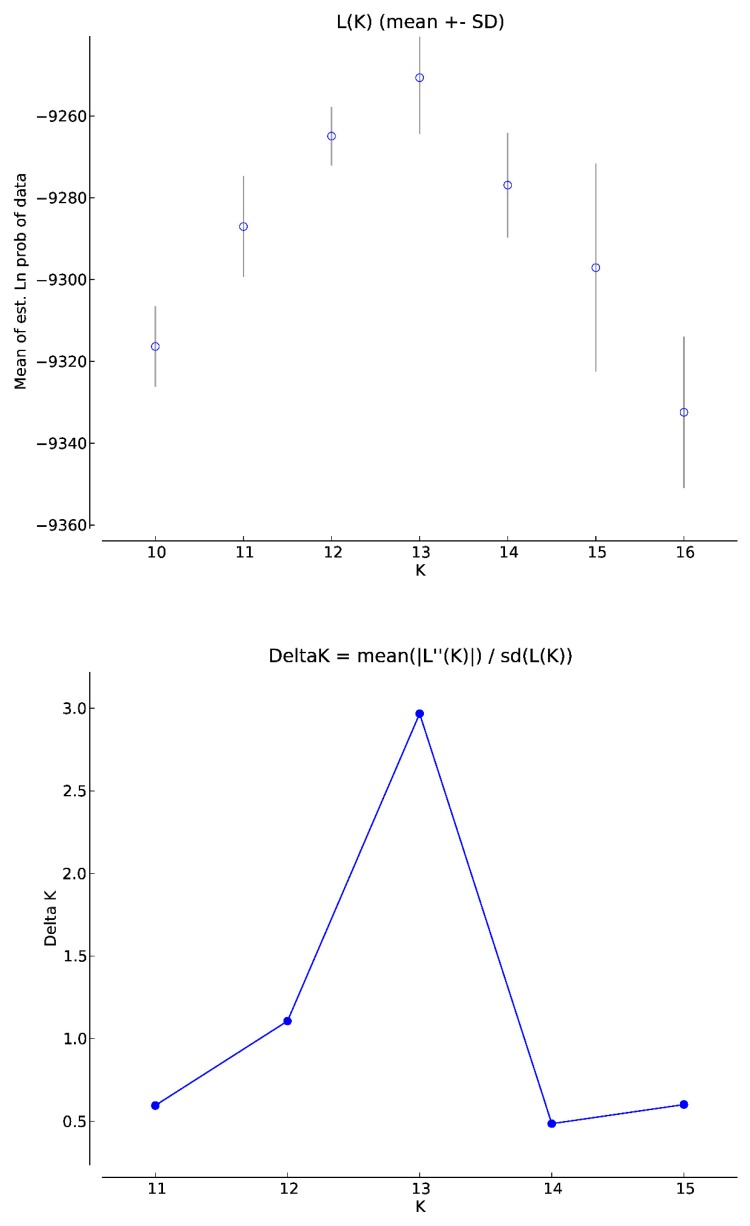
K = 13 was obtained by the highest likelihood and ΔK method.

**Figure 2:**
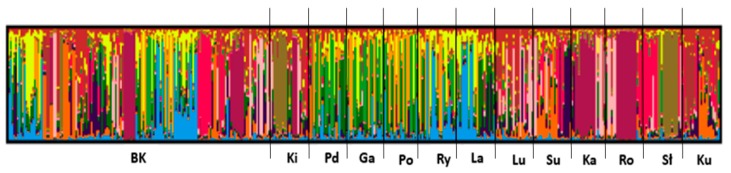
Visualization of population structure in examined geese populations obtained through CLUMPP software. Vertical lines represent individuals and each color represents one cluster (BK—White Kołuda^®^ goose, Ki—Kielecka, Pd—Podkarpacka, Ga—Garbonosa, Po—Pomerian, Ry—Rypinska, La—Landes, Lu—Lubelska, Su—Suwalska, Ka—Kartuska, Ro—Romanska, Sł—Slovacka, Ku—Kubanska).

**Figure 3:**
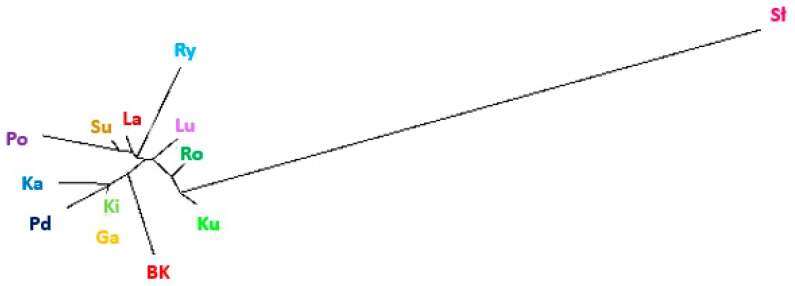
Genetic relations among 13 examined goose breeds with the use of the neighbor joining tree based on genetic distance (BK—White Kołuda^®^ goose, Ki—Kielecka, Pd—Podkarpacka, Ga—Garbonosa, Po—Pomerian, Ry—Rypinska, La—Landes, Lu—Lubelska, Su—Suwalska, Ka—Kartuska, Ro—Romanska, Sł—Slovacka, Ku—Kubanska).

We used the Structure software, which is based on the Bayesian model, clustering algorithms of multi-locus genotypes to identify the population structure and the pattern of admixture within populations. Corresponding to the number of goose breeds, the highest likelihood was obtained for K = 13 (Figure 1). Division of runs in K = 13 was 10/10 (10 runs presented very similar results) and the similarity score obtained was 0.899. The results of the analysis for all the populations, generated in CLUMPAK software, are shown in Figure 2 and Table 4. The graph shows a highly mixed genotype of all the examined geese (Figure 2). The data show no clear and distinct clusters and the populations are defined with a high level of admixture. There are individuals assigned to all 13 clusters in the White Kołuda^®^ breed, suggesting that it cannot be distinguished from other breeds on the basis of microsatellite markers (Figure 2). The genetic distances between them generated the neighbor joining dendrogram (Figure 3), showing that the Slovacka breed is in the most distant position from other breeds while the closest relationships are among Kielecka and Garbonosa breeds.

We have also conducted a F_ST_ pairwise analysis (Table 5). The F_ST_ values between the groups of examined geese were varied. Significantly low F_ST_ values was observed between Pomerian and Rypinska geese, the index value was 0.003; also between Pomerian and Garbonosa the value of F_ST_ pairwise was 0.005, which indicates low genetic differentiation. The F_ST_ values among the population of Landes and Romanska was high (0.261) and significantly greater than other breeds. Moreover Rypinska and Romanska was also significantly larger (0.233). The rest of the F_ST_ index among breeds was characterized by medium values. We presume Romanska geese perform the highest differentiation among those examined breeds.

**Figure 4:**
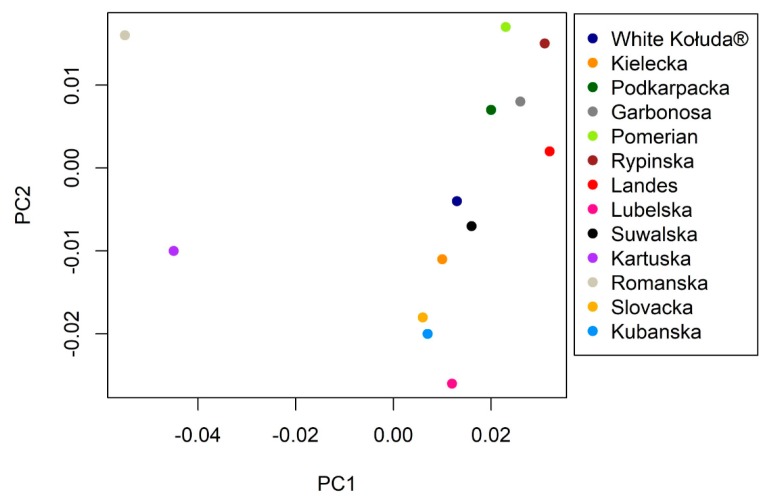
Principal component analysis (PCA) of 13 goose breeds from 14 microsatellite loci.

Principal component analysis (PCA) is shown plotted on Figure 4, revealing the high divergence of the Romanska breed from other geese; also Kartuska is genetically distanced, which corresponds to the F_ST_ pairwise analysis. The rest of the breeds are grouped close to each other.

## 4. Discussion

Waterfowl represent a diverse group of birds which are intensively examined worldwide. Investigation of the genetic diversity of goose breeds using modern methods of molecular genetics can be a great support in the development of goose breeding programs and conservation of old local breeds’ purity. The identification of breeds’ genetic diversity and genetic uniqueness is applicable in breeding of animals and is becoming essential nowadays.

We analyzed 14 microsatellite loci tested previously and proved to be polymorphic: in Polish local breeds (eight microsatellite loci: Bca µ1, TTUCG5, CKW21, Bca µ9, Bca µ8, CAUD-G013, CAUD-G007, Aal µ1, CAUD-G012) and European breeds (five microsatellite loci: Ans02, Ans18, Ans25, Afa35, Ans07) [5,7,17,24,25]. Moreover, this was the first analysis including biological material of the White Kołuda^®^ goose. Generally, we observed a similar level of polymorphism in analyzed loci as previously reported in literature. In our study, the most polymorphic loci were TTUCG5 and CKW21; we observed 16 and 15 different alleles in all analyzed breeds. Accordingly, these loci displayed the highest PIC values. Similar results were obtained by Parada et al. [5], who analyzed eight Polish breeds of geese and observed 18 and 19 alleles at TTUCG5 and CKW 21, respectively. Andres et al. (2011) detected a higher number of alleles in CKW21 in the Zatorska breed, but the highest PIC and H_o_ were observed in Bca µ1 locus, which in our study displayed intermediate polymorphism. The same number of alleles (four) were observed in Bca µ8 according to our studies and Andres et al. [7], though compared to other analyzed markers it did not show a high level of allele content. Furthermore, Afa35 was the most polymorphic microsatellite loci in Bean geese according to Kleven et al. [25], with seven alleles in the Norwegian population. In our populations we observed even higher polymorphism of this marker with 10 different alleles. However, eight of them were observed in the White Kołuda^®^ breed, while in Rypinska, Pomerian, Landes, Podkarpacka, and Slovacka only one allele was monomorphic. However, the number of White Kołuda^®^ individuals was higher than other breeds, so that the probability of private alleles increases. According to Barker [26], the microsatellites with at least four alleles in loci are considered to be useful in evaluating the genetic diversity in order to reduce the standard mistakes of distance estimation. In our study, the lowest number of alleles was observed in CAUD-G007 (three alleles), which suggests that this marker is not useful for diversity investigations. Apart from CKW47, which was monomorphic, all remaining markers had more than three alleles.

It is accepted that the value of polymorphism information content should be above 0.5, which indicates the most informative markers in population. Moderately informative markers are those between PIC value 0.25–0.5, while low informative are those under 0.25 [22]. PIC in our research varied from 0.165 (CAUD-G007) to 0.813 (TTUCG5), while in Botstein’s interpretation, six of the analyzed loci are highly informative markers (TTUCG5, CKW21, Bca µ8, Aal µ1, CAUD-G012, Ans25,), six loci are medium informative markers (Bca µ1, Bca µ9, Ans02, Ans18 CAUD-G013, Ans07), and two characterize a low PIC value (CAUD-G007, Afa35). According to Andres et al. [7], 15 loci of Zatorska geese exceeded 0.25 PIC value, while in the research of Parada et. al. [5] on Polish conservative flocks, four of the analyzed loci were highly informative, six were moderately, and four remained to be less informative. Corresponding to Li et al. [14], who carried out the experiment on Chinese geese, 13 out of 31 markers were medium informative.

One of the most important coefficients estimating genetic variability in the population is heterozygosity. The average observed heterozygosity of the populations was 0.361, ranging from 0.119 (Afa35) to 0.721 (TTUCG5), while the expected overall polymorphic loci was 0.447, which corresponds to results obtained by Andres et al. [7] in the Zatorska geese (mean H_o_ = 0.35, H_e_ = 0.38) and to the wild graylag geese where the observed and expected heterozygosity were 0.36 and 0.42, respectively [8]. In the Slovak geese, the microsatellite with the highest H_o_ value is TTUCG5, as was obtained in our study [17]. The mean observed heterozygosity across the populations of analyzed geese vary from 0.479 to 0.280, which is similar to other European geese (0.374–0.483), the same as the expected heterozygosity, which ranged from 0.305 to 0.638, remaining similar to European and Chinese geese populations [10,24]. To conclude, the analyzed panel of microsatellite loci can be considered as sufficient to assess the genetic diversity and structure of geese populations. Moreover, marker TTUCG5C had the highest genetic diversity among all chosen microsatellites in our study.

**Figure 5:**
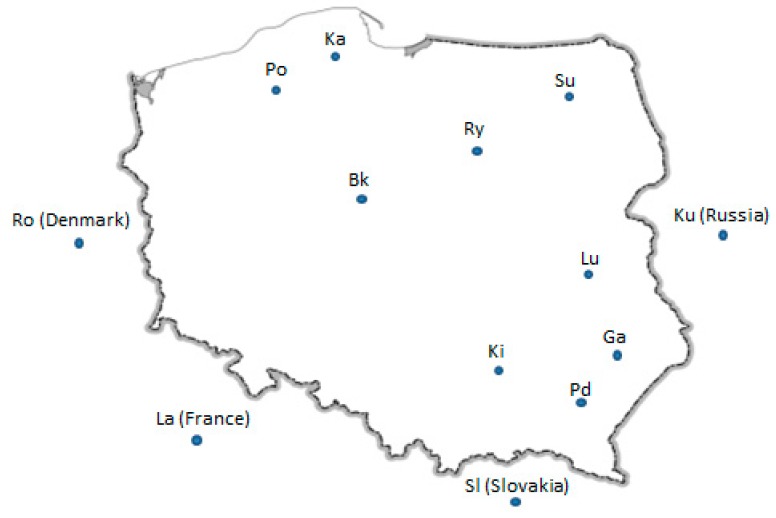
Map showing origin of occurrence of indigenous geese and four foreign breeds kept in Poland [6].

The Structure analysis revealed that the most probable number of subpopulations in analyzed breeds is 13 (Figure 1), which is in line with our classification of analyzed geese. Most of the examined individuals displayed admixed genotypes regardless of the number of groups considered in the experiment. We did not observe clear clusters among analyzed breeds of geese, which may indicate a high level of differentiation. According to the results, we can presume that the genetic distance between tested goose breeds is low. Although according to Stracture (Figure 3), the Slovak goose seems to be the most divergent, other analyses (PCA (Figure 4), F_ST_ pairwise (Table 5)) do not confirm this. This may be due to different approaches and different calculations systems used in this application. Our findings correspond to those of Pellegrino et al. [24], who carried out research on the greylag goose, indicating the presence of highly mixed genotypes. Our research is the first attempt at analysis of the relationship between goose breeds kept in Poland using Structure software. According to the F_ST_ pairwise and PCA analysis, the highly distanced breeds are Romanska and Kartuska. The origin of the Romanska goose is Denmark, which could explain the high differentiation between it and most of the populations compared. Geese that are characterized by a relevant phenotype consistent with the family pattern are classified as a specific breed and entered into the book of breeding animals and the national protection program of genetic resources by experienced poultry inspectors. Therefore, currently the selection of geese in Poland relies on phenotype and no genetic selection is carried out. The most important traits considered during selection are body weight, meatiness, and meat quality; thus inbreeding could be a common phenomenon among goose breeds. Close relationships among the populations could be possible, associated with the historical relations and geographical distribution [14]. The majority of goose breeds in Europe, as in Poland, originate from *Anser anser* (Kartuska, Rypinska, Suwalska, Pomerian, Lubelska, Kielecka, Podkarpacka, Romanska, Slovacka); however, two breeds have a different ancestor—*Anser cygnoides* (Garbonosa and Kubanska) [27] and their names were given according to the place of origin. Geese from the indigenous flocks kept by the National Institute of Animal Production are the only representatives of this population and they do not occur in other regions of Poland [28]. Moreover, geese are divided into North and South groups depending on the region of origin. Northern geese include Pomerian, Kartuska, Rypinska, and Suwalska, while the Southern are Lubelska, Kielecka, and Podkarpacka. Foreign geese include Romanska, Slovacka, Landes, and Kubanska (Figure 5). Historical data are not well documented; however, we obtained the PhD thesis of Wrzaszcz [29] and the National Research Institute of Animal Production website, where the data are included [6]. Wrzaszcz indicated occurrence of division into groups derived from *Anser anser* and *Anser cygnoides* [29]. Another classification is based on morphological traits; thus geese are divided into heavy and light geese depending on their body weight. Sothern geese, Garbonosa, and Kubanska are characterized as light weight, while the Northern and Romanska geese are heavy geese. Slovacka’s weight is defined as medium compared to the rest [27]. This may explain the separateness of the Slovacka goose compared to the other breeds revealed in our study by genetic distance (Figure 3). Moreover, the examined geese show differences in plumage. Kielecka, Lubelska, Pomerian, Slovacka, Romanska, and White Kołuda^®^ are reported to have whole body white plumage. White and speckled grey or brown plumage occur in Garbonosa, Kartuska, Podkarpacka, Rypinska, Suwalska, Kubanska, and Landes [6]. However we presume that color of plumage does not affect our results.

## 5. Conclusions

The White Kołuda^®^ goose constitutes 98% of the goose population bred in Poland [30] and was officially recognized as a breed in 2012. It is characterized by very good meatiness and exceptional feather quality; therefore, products obtained from it are marked with the trademark “Koludzka White”. The results of our analyses indicate that this is not possible with the use of STR markers. However, we do not rule out the possibility of confirming this with usage of more microsatellite markers or a larger group of geese. Moreover, some of the results are ambiguous, thus we did not draw far-reaching conclusions. Therefore, we will look for other genetic markers based on modern molecular techniques such as mtDNA analysis or genotyping by synthesis (NGS). It is possible that as breeding of the White Kołuda^®^ goose progresses, the diversity within it will decrease, the diversity among breeds will increase, and this breed will create a separate genetic population. On the other hand, the high variability within the breed is a favorable phenomenon, indicating the high selection potential of this breed. In conclusion, we carried out the first analysis of the genetic variability of geese maintained in Poland based on Structure analysis. It has indicated that geese bred in Poland do not form separate populations in genetic terms and are characterized by a high level of mixing genotypes at the STR locus.

## Figures and Tables

**Table 1:** Characteristics of 15 polymorphic microsatellite loci in analyzed goose population.

Locus	GeneBank Accesion No.	Source Species	Repeat Motif of Sequenced Clone	Primer Sequence (5′–3′)	Temp. of Amplification
Bca µ1	AF025889	*Branta canadensis*	(TA)_15_ (CA)_10_	F: TGCTTTTTACCCCCAGTGTTCT R: AGAATCTGCTATATTATTTCCAGCTC	61
TTUCG5	U66093	*Branta canadensis*	TCTAT	F: GGGTGTTTTCCAACTCAG R: CACTTTCCTTACCTCATCTT	61
CKW21	-	*Anser cygnoides*	(TTA)_10_	F: CAAGGTAGTCATAAACCCAGAACA R: ACAAAACTAATGGCAGGAAAC	62
Bca µ9	AF025897	*Branta canadensis*	(CA)_9_	F: CCCAGTTCCTCTCATTCTCCTT R: AAACAGGGAGGTGAAAGT	61
Bca µ8	AF025896	*Branta canadensis*	(CA)_8_	F: CCCAAGACTCACAAAACCAGAAAT R: ATGAAAGAAGAGTTAAACGTGTGCAA	58
Ans02	EU833383	*Anser anser*	(AG)_17_	F: TTCTGTGCAGGGGCGAGTT R: AGGGAACCGATCACGACATG	58
Ans18	EU833373	*Anser anser*	(AC)_12_ AT(AC)_6_	F: GTGTTCTCTGTTTATGATATTAC R: AACAGAATTTGCTTGAAACTGC	58
Ans25	EU833378	*Anser anser*	(GT)_18_	F: CACTTATTAATGGCACTTGAAA R: GTTCTCTTGTCACAACTGGA	58
CAUD-G013	AY493258	*Anas platyrhynchos domesticus*	(AC)_9_	F: ACAATAGATTCCAGATGCTGAA R: ATGTCTGAGTCCTCGGAGC	61
CAUD-G007	AY493252	*Anas platyrhynchos domesticus*	(CAG)_5_ (GCA)_5_	F: ACTTCTCTTGTAGGCATGTCA R: CACCTGTTGCTCCTGCTGT	61
Aal µ1	U63689	*Anser albifrons*	TG	F: CATGCGTGTTTAAGGGGTAT R: TAAGACTTGCGTGAGGAATA	55
Afa35	KT698203	*Anser fabalis*	(AGAC)_10_ (AGAA)_7_	F: ACCCTGCCAGATCTCTTGTC R: GCCCATTTTTCTAAAGAAGATGCC	60
CAUD-G012	AY493257	*Anas platyrhynchos domesticus*	(AC)_10_	F: ATTGCCTTTCAGTGGAGTTTC R: CGGCTCTAAACACATGAATG	57
CKW47	AY790335	*Anser cygnoides*	(T)_8_(TG)_7_	F: AACTTCTGCACCTAAAAACTGTCA R:TGCTGAGGTAACAGGAATTAAAA	62
Ans07	EU833363	*Anser anser*	(CA)_11_	F: GACTGAGGAACTACAATTGACT R: ACAAAGACTACTACTGCCAAG	58

**Table 2:** Summary of genetic variation at 14 microsatellite loci.

Population	BK	Ki	Pd	Ga	Po	Ry	La	Lu	Su	Ka	Ro	Sł	Ku	Mean
N	152	22	22	22	20	22	23	22	22	20	21	23	22	-
Na	6929	4,857	3214	2714	2786	2714	2929	3714	4000	4714	5000	3929	3857	3951
Ne	2348	2446	1870	1805	1928	1800	1746	2147	2191	2822	3041	2420	2056	2202
Ho	0.362	0.308	0.334	0.328	0.329	0.295	0.280	0.396	0.409	0.479	0.415	0.401	0.354	0.361
He	0.490	0.479	0.384	0.365	0.369	0.340	0.305	0.423	0.479	0.620	0.638	0.478	0.442	0.447
FST	0.073	0.074	0.077	0.078	0.077	0.078	0.080	0.076	0.074	0.069	0.068	0.074	0.075	0.075

Notes: N—number of individuals; Na—No. of different alleles; Ne—No. of effective alleles; H_o_—observed heterozygosity; H_e_—expected heterozygosity.

**Table 3:** Summary of fixation index value and polymorphism information content at 14 microsatellite loci.

Populations	Bca µ1	TTUCG5	CKW21	Bca µ9	Bca µ8	Ans02	Ans18	Ans25	CAUD-G013	CAUD-G007	Aal µ1	Afa35	CAUD-G012	Ans07	Mean
Fst	0.077	0.095	0.068	0.106	0.117	0.140	0.300	0.084	0.089	0.151	0.061	0.156	0.139	0.127	0.122
PIC	0.381	0.813	0.686	0.447	0.561	0.299	0.331	0.633	0.444	0.165	0.551	0.191	0.546	0.433	0.463
Ho	0.366	0.721	0.620	0.358	0.400	0.235	0.045	0.600	0.430	0.122	0.519	0.119	0.437	0.075	0.361
He	0.386	0.754	0.664	0.446	0.557	0.283	0.252	0.623	0.506	0.138	0.560	0.017	0.507	0.415	0.447

**Table 4:** Average membership coefficient of each predefined population in each of the 13 clusters.

Population	Inferred Clusters													Number of Individuals
	1	2	3	4	5	6	7	8	9	10	11	12	13	
BK	0.057	0.09	0.066	0.087	0.035	0.077	0.068	0.077	0.07	0.119	0.076	0.097	0.08	132
Ki	0.017	0.051	0.022	0.053	0.31	0.109	0.087	0.066	0.078	0.022	0.024	0.123	0.038	22
Pd	0.128	0.131	0.277	0.155	0.049	0.014	0.051	0.042	0.017	0.071	0.04	0.003	0.021	22
Ga	0.178	0.13	0.335	0.131	0.014	0.018	0.036	0.031	0.025	0.078	0.013	0.002	0.009	22
Po	0.259	0.112	0.317	0.127	0.008	0.01	0.016	0.039	0.01	0.078	0.011	0.002	0.01	20
Ry	0.212	0.17	0.097	0.159	0.027	0.008	0.022	0.031	0.015	0.222	0.022	0.002	0.013	22
La	0.059	0.178	0.056	0.181	0.051	0.015	0.034	0.089	0.033	0.278	0.012	0.003	0.011	23
Lu	0.017	0.043	0.023	0.044	0.025	0.264	0.279	0.077	0.118	0.022	0.046	0.003	0.039	22
Su	0.015	0.038	0.016	0.039	0.04	0.135	0.033	0.223	0.074	0.019	0.216	0.007	0.146	22
Ka	0.009	0.031	0.017	0.033	0.047	0.173	0.033	0.049	0.062	0.016	0.029	0.487	0.015	20
Ro	0.007	0.024	0.013	0.027	0.009	0.176	0.024	0.017	0.031	0.011	0.025	0.559	0.076	21
Sł	0.009	0.025	0.031	0.021	0.43	0.071	0.017	0.025	0.02	0.018	0.016	0.003	0.316	23
Ku	0.017	0.027	0.031	0.023	0.043	0.026	0.152	0.118	0.261	0.024	0.136	0.007	0.137	22

BK—White Kołuda^®^ goose, Ki—Kielecka, Pd—Podkarpacka, Ga—Garbonosa, Po—Pomerian, Ry—Rypinska, La—Landes, Lu—Lubelska, Su—Suwalska, Ka—Kartuska, Ro—Romanska, Sł—Slovacka, Ku—Kubanska.

**Table 5:** The value of the fixation index (F_ST_) pairwise between all groups of geese.

	BK	Ki	Pd	Ga	Po	Ry	La	Lu	Su	Ka	Ro	Sł	Ku
**BK**	0.000												
**Ki**	0.008	0.000											
**Pd**	0.022	0.058	0.000										
**Ga**	0.019	0.055	0.009	0.000									
**Po**	0.026	0.067	0.019	0.005	0.000								
**Ry**	0.020	0.068	0.033	0.010	0.003	0.000							
**La**	0.023	0.047	0.064	0.032	0.052	0.009	0.000						
**Lu**	0.030	0.017	0.067	0.090	0.095	0.099	0.091	0.000					
**Su**	0.033	0.037	0.088	0.094	0.104	0.090	0.104	0.058	0.000				
**Ka**	0.106	0.098	0.155	0.161	0.166	0.190	0.204	0.152	0.130	0.000			
**Ro**	0.163	0.148	0.208	0.220	0.208	0.233	0.261	0.208	0.183	0.051	0.000		
**Sł**	0.036	0.019	0.088	0.088	0.109	0.099	0.083	0.049	0.043	0.120	0.187	0.000	
**Ku**	0.017	0.014	0.058	0.064	0.089	0.084	0.080	0.027	0.025	0.118	0.191	0.024	0.000

## Supplementary Materials

The following are available online at https://www.mdpi.com/2076-2615/9/10/737/s1, Table S1: Genotyping data, Table S2: Number of different of alleles at 14 microsatellite loci.
